# Inflammatory Markers and Obstructive Sleep Apnea in Obese Children: The NANOS Study

**DOI:** 10.1155/2014/605280

**Published:** 2014-06-01

**Authors:** Alex Gileles-Hillel, María Luz Alonso-Álvarez, Leila Kheirandish-Gozal, Eduard Peris, José Aurelio Cordero-Guevara, Joaquin Terán-Santos, Mónica Gonzalez Martinez, María José Jurado-Luque, Jaime Corral-Peñafiel, Joaquin Duran-Cantolla, David Gozal

**Affiliations:** ^1^Section of Sleep Medicine, Department of Pediatrics, Comer Children's Hospital, Pritzker School of Medicine, Biological Sciences Division, The University of Chicago, 5721 S. Maryland Avenue, Chicago, IL 60637, USA; ^2^Sleep Unit, Instituto Carlos III.CIBERES, Spain; ^3^CIBER of Respiratory Diseases, Instituto Carlos III.CIBERES, Spain; ^4^Hospital Universitario de Burgos (HUBU), Burgos, Spain; ^5^Hospital Universitario Valdecilla, Santander, Spain; ^6^Hospital Val d'Hebron, Barcelona, Spain; ^7^Hospital San Pedro de Alcantara, Caceres, Spain; ^8^Servicio de Investigación, Hospital Universitario Araba (BioAraba), Universidad del País Vasco (UPV/EHU), Vitoria, Spain

## Abstract

*Introduction.* Obesity and obstructive sleep apnea syndrome (OSA) are common coexisting conditions associated with a chronic low-grade inflammatory state underlying some of the cognitive, metabolic, and cardiovascular morbidities. *Aim.* To examine the levels of inflammatory markers in obese community-dwelling children with OSA, as compared to no-OSA, and their association with clinical and polysomnographic (PSG) variables. *Methods.* In this cross-sectional, prospective multicenter study, healthy obese Spanish children (ages 4–15 years) were randomly selected and underwent nocturnal PSG followed by a morning fasting blood draw. Plasma samples were assayed for multiple inflammatory markers. *Results.* 204 children were enrolled in the study; 75 had OSA, defined by an obstructive respiratory disturbance index (RDI) of 3 events/hour total sleep time (TST). BMI, gender, and age were similar in OSA and no-OSA children. Monocyte chemoattractant protein-1 (MCP-1) and plasminogen activator inhibitor-1 (PAI-1) levels were significantly higher in OSA children, with interleukin-6 concentrations being higher in moderate-severe OSA (i.e., AHI > 5/hrTST; *P* < 0.01), while MCP-1 levels were associated with more prolonged nocturnal hypercapnia (*P* < 0.001). *Conclusion.* IL-6, MCP-1, and PAI-1 are altered in the context of OSA among community-based obese children further reinforcing the proinflammatory effects of sleep disorders such as OSA. This trial is registered with ClinicalTrials.gov NCT01322763.

## 1. Introduction

Sleep-disordered-breathing (SDB) is a group of common disorders characterized by habitual snoring along with varying degrees of gas exchange alterations and sleep fragmentation [1]. Obstructive sleep apnea (OSA) is the most prevalent of those disorders affecting 1–4% of children with a peak incidence around 2–8 years [2]. In recent years, it has become apparent that the frequency of OSA is markedly increased by the concurrent presence of obesity [3] and the coexistence of these 2 conditions has been linked to a higher risk for development of end-organ morbidities, including neurocognitive and behavioral impairments and cardiovascular and metabolic dysfunction [4–8]. In addition to increased oxidative stress, activation and propagation of inflammatory pathways in the context of immune dysregulation have been implicated in the deleterious consequences of OSA [9, 10], with the cumulative evidence strongly supporting the concept that pediatric OSA is a chronic, low grade inflammatory condition [11–16]. In this context, it is now recognized that OSA causes, albeit not always, systemic elevation in the levels of inflammatory mediators, such as CRP, TNF*α*, IL-6, and INF-*γ* [17–23], and the concomitant reduction of anti-inflammatory substances, such as IL-10, thereby tilting the balance toward a heightened proinflammatory state [24].

Similarly, obesity has long been recognized as an indolent and persistent inflammatory condition in which the sustained activity of such processes promotes the occurrence of insulin resistance and vascular dysfunction [25–29]. OSA and obesity frequently coexist in children and have been assumed to interact and promote each other [30–32].

However, the potential contributions of OSA to the proinflammatory profile of obese children have not been critically delineated, particularly considering the incongruent inflammatory phenotypes that have been previously reported in obese children [33]. Thus, we hypothesized that community-recruited obese children with OSA would display significant differences in their plasma levels of specific biomarkers, including inflammatory markers. The aim of the present study was to assess and better delineate the potential effects of perturbed sleep, such as occurs in OSA, on a panel of inflammatory cytokines and adipokines in a large cohort of obese children.

## 2. Subjects and Methods

204 obese children (ages 4–15 years) were recruited from the community in a multicenter prospective study in Spain—the NANOS study. A detailed account of the study design is described elsewhere [34]. Briefly, obese children were prospectively enrolled through primary care centers across Spain during 2007–2010 and were randomly selected to participate in the study. The study was approved by a human subject committee in each of the participating centers and is in accordance with the STROBE statement. The study was registered at ClinicalTrials.gov under NCT01322763. Informed consent was obtained from each subject or legal guardian, and assent was obtained from children above 12 years old. Data was coded so each investigator in the research network was blinded to subjects' personal information and thus ensuring confidentiality. Samples and data from subjects included in this study were provided by the Basque Biobank for research OEHUN (http://www.biobancovasco.org/) and were processed following standard operating procedures with appropriate approvals from the Ethical and Scientific Committees.

The general medical and sleep histories were obtained from all participating children and the parents filled a validated Spanish version of the Pediatric Sleep Questionnaire (PSQ) [35]. Every child then underwent a thorough medical examination followed by an overnight sleep study (PSG). The morning after the PSG, blood was drawn in fasting conditions.

### 2.1. Overnight Polysomnography

PSG tests were conducted in a sleep laboratory under standardized conditions. The studies were scored, after removal of movement and technical artifacts, according to the standard criteria defined by the American Academy of Sleep Medicine (AASM) [36]. Briefly, obstructive sleep apnea was defined as cessation of airflow with continued chest wall and abdominal movements for the duration of at least of two breaths. Hypopnea was defined as a decrease in nasal flow greater than 50%, corresponding to at least 4% decrease in the oxygen saturation (SpO_2_) as measured by pulse oximetry and/or terminated by a 3-second EEG arousal. The obstructive apnea-hypopnea index (AHI) was defined as the number of apneas and hypopneas per hour of total sleep time (TST). The obstructive respiratory disturbance index (RDI) was calculated from the number of respiratory-effort-related arousals and the number of apneas and hypopneas per hour of TST. Children with OSA were defined as those having RDI ≥ 3/hr of TST in accordance with the clinical practice guidelines in Spain [37]. In addition, nadir and mean SpO_2_, as well total sleep time during which SpO_2_ is below 90% or end-tidal CO_2_ > 50 mmHg   occurred, were recorded. Oxygen desaturation index (ODI) was defined as the number of desaturation events ≥ 4% per hour of TST. Of note, in our subanalyses we defined moderate-to-severe OSA, as the presence of obstructive AHI > 5/hrTST.

### 2.2. Inflammatory Mediator Assays

Plasma was separated from the whole blood morning samples drawn from each child and stored in −80°C until assay. Commercially available ELISA kits specific for each cytokine were used to measure levels of IL-6, IL-18, MCP-1, adiponectin, MMP-9, apelin C, leptin (all individual kits from RayBiotech, Inc., Norcross, GA, USA), adropin (Peninsula laboratories LLC, San Carlos, CA, USA), osteocrin (MyBioSource, San Diego, CA, USA), and PAI-1 (Assaypro LLC, St. Charles, MO, USA). Assays were performed according to manufacturers' recommendations.

### 2.3. Statistical Analysis

Descriptive data for continuous variables are presented as means ± standard deviation (SD) and for categorical variables as percentages or ratios. Analyses for comparisons between clinical and laboratory values among OSA and no-OSA groups were performed using Student's* t*-tests for continuous and *χ* ^2^ tests for categorical variables followed by Fisher exact tests. Group comparisons were conducted using one-way ANOVA followed by Bonferroni correction for multiple comparisons. Pearson's correlation was used to compare between the marker levels and clinical parameters. Multivariate linear regression analysis was applied to assess relationships of significantly different markers between the two groups of children. Statistical significance was assumed at two-tailed *P* < 0.05. Statistical analyses were performed using SPSS software (version 21.0; SPSS Inc., Chicago, IL).

## 3. Results

### 3.1. Demographic Data

204 obese children from the community (ages 4–15 years) were recruited from the NANOS study, 111 boys and 93 girls, all fulfilling obesity criteria, that is, BMI above the 95% for age and gender [38]. The prevalence of OSA in this group of obese children was 36.7%. The 2 groups of children, those with (OSA) and without OSA (no-OSA), had similar demographic and anthropometric characteristics (Table 1).

### 3.2. Sleep Studies

PSG findings are summarized in Table 2 for the 2 groups. As would be anticipated from the OSA and no-OSA category allocation, most of the PSG variables differed, and most particularly for respiratory parameters and the number of arousals from sleep (Table 2). In contrast, there were no significant differences in either the total duration of sleep and total time in bed (Table 2). These findings support the idea that disruption of sleep architecture, that is, sleep fragmentation, rather than sleep deprivation, is the salient sleep perturbation among children with OSA [4].

### 3.3. Plasma Inflammatory Mediators in Obese Children: OSA versus No-OSA

Among the inflammatory markers included in the present study, 2 markers were significantly higher in the OSA group, namely, PAI-1 (Table 3; *P* = 0.01) and MCP-1 (Table 3; *P* = 0.03). In a subset of children with more severe OSA (i.e., AHI > 5/hrTST), significantly higher levels of IL-6 emerged (*P* = 0.009; Table 3). In addition, MCP-1 levels of ≥30 pg/mL and PAI-1 of ≥3.3 ng/mL conferred a modestly higher risk of OSA (OR = 2, CI_95%_ = 1.1–3.6, *P* = 0.02; OR = 1.8, CI_95%_ = 1–3.2, *P* = 0.04, resp.).

To further examine the global contribution of inflammatory markers to the overall inflammatory state of each child, we constructed a cumulative “inflammatory score” (IS), whereby each marker was standardized using* z*-score transformation. The IS was then calculated by summarizing all the individual* z* scores. Please note that the* z* scores for adiponectin and adropin were calculated and multiplied by −1, since their plasma levels have been reported to decrease in states of increased inflammation and obesity. The IS was significantly higher in the OSA as compared to no-OSA groups (Table 3; *P* = 0.04).

No differences in inflammatory marker levels emerged between boys and girls in the full cohort, except for higher plasma levels of leptin among girls (17.1 versus 21.3 ng/mL, *P* < 0.001). Of note, girls had slightly lower baseline and mean SpO_2_ levels during the PSG (mean difference ~0.5%, *P* = 0.01) and a trend toward lower BMI% (96.8 versus 96.7%, *P* = 0.05).

### 3.4. Correlation Analyses

First, we examined whether the various biomarkers were associated with both PSG-derived measures and anthropometric measurements in the full cohort (*n* = 204; Table 3). Higher MCP-1 levels correlated with ODI (*r* = −0.171; *P* = 0.02), with TCO_2_ > 50 (*r* = 0.352; *P* < 0.001) and with peak CO_2_ levels (*r* = 0.168; *P* = 0.02). These correlations remained statistically significant after adjusting for age, gender, and BMI. Leptin was positively associated with higher BMI, older age, female gender, and shorter sleep duration, and such associations remained significant even after adjusting for other confounders (*P* ≤ 0.006). Higher leptin levels were also associated with lower sleep efficiency (after adjusting for age), but this effect disappeared when adjusted for BMI. Adiponectin was negatively correlated with age and BMI (*r* = −0.3; *P* < 0.001), while age-adjusted adiponectin levels were borderline associated with BMI (*P* = 0.054).

Additionally, IS had a strong positive correlation with BMI (*r* = 0.241, *P* < 0.001), neck circumference (*r* = 0.226, *P* < 0.001), age (*r* = 0.154, *P* = 0.01), and TCO_2_ > 50 (*r* = 0.294, *P* < 0.001) and was inversely associated with TST (*r* = −0.172, *P* = 0.007) and sleep efficiency (*r* = −0.142, *P* = 0.026). In a linear regression model that included all of the above variables that had significant correlations with IS, BMI and TCO_2_ > 50 independently predicted higher IS (*β* = 0.296, *P* = 0.001; *β* = 0.360, *P* < 0.001).

Next, we examined whether any of the specific markers was potentially useful in predicting clinically relevant components of sleep-disordered breathing among the 75 children with OSA, that is, sleep fragmentation, intermittent hypoxemia, and hypercapnia. Pearson correlation coefficients (PCC) are presented and only the results that remained statistically significant after age adjustment are presented below, given the considerable changes in marker levels as a function of age (Table 4). Significant associations were observed for MCP-1 levels and ODI (*r* = −0.276; *P* = 0.01), Nadir SpO_2_ (*r* = 0.232; *P* = 0.02), and TCO_2_ > 50 (*r* = 0.412; *P* < 0.001). MCP-1 association with ODI remained significant after adjusting for age, sex, and BMI. Leptin was associated with lower TST (*r* = −0.413, *P* < 0.001). Adropin was associated with lower total time in bed (*r* = −0.363; *P* = 0.001), baseline SpO_2_ (*r* = −0.471; *P* < 0.001), peak CO_2_ (*r* = −0.389; *P* = 0.001), and TCO_2_ > 50 (*r* = −0.335; *P* = 0.007). MMP-9 was associated with lower total time in bed (*r* = −0.310; *P* = 0.007) and with higher TCO_2_ > 50 (0.273; *P* = 0.03). Finally, apelin C exhibited a strong positive correlation with TCO_2_ > 50 (*r* = 0.511; *P* < 0.001).

In a multivariate analysis that included all the marker levels in the OSA group aiming at correcting for intermarker correlations, age-adjusted MCP-1 levels remained the only inflammatory mediator that independently predicted TCO_2_ > 50 (*β* = 0.322, *P* = 0.03). Furthermore, age-adjusted leptin levels in the OSA group independently predicted lower TST (*β* = −0.252, *P* = 0.04). Inflammatory score (IS) was correlated in the OSA group with higher TCO_2_ > 50 (*r* = 0.359, *P* = 0.002) and had borderline association with neck circumference (*r* = 0.213, *P* = 0.049). Only higher TCO_2_ > 50 independently predicted higher IS (*β* = 0.356, *P* = 0.003) in the OSA group in a model that included age, BMI, and neck circumference.

## 4. Discussion

Current findings provide incremental evidence that the presence of OSA operates as an independent contributor to the increased systemic inflammation that occurs in obese children. Our data indicate that the levels of two blood markers, namely, PAI-1 and MCP-1, were increased among obese children with OSA, such that plasma concentrations of MCP-1 > 30 pg /mL and PAI-1 > 3.3 ng/mL provide reliable prediction on the presence of OSA. In addition, in a subset of obese children with moderate-to-severe OSA, IL-6 levels were also significantly higher. Furthermore, the overall inflammatory status, as inferred from the inflammatory score (IS), an arbitrary additive summation of the relative levels of all the current markers assayed in this study, was significantly increased in the OSA group, indicating heightened overall inflammatory load in OSA. Interestingly, IS also exhibited significant associations with BMI and total sleep time and efficiency as well as with the duration of hypercapnia.

Before discussing the potential implications of our findings, we will initially focus on those 3 inflammatory mediators that were markedly elevated in the OSA group, MCP-1, PAI-1, and IL-6. Monocyte chemoattractant protein 1 (MCP-1) is a central member of the C-C chemokine superfamily responsible for attracting mononuclear cells to inflammatory sites [39]. MCP-1 increases with obesity, plays a role in recruiting macrophages into adipose tissue in adult obese patients [40–42], and is associated with insulin resistance and with type 2 diabetes [43]. This cytokine, which is also highly expressed in the inflamed vasculature, is a potent attractor of lipid-activated monocytes involved in the inflammatory signaling cascade related to vascular dysfunction, atherosclerosis, and cardiac events [44, 45]. In children, there is also evidence that MCP-1 increases with obesity [46, 47]. In the context of OSA, MCP-1 elevations have been reported in adult patients, and treatment with CPAP reduced MCP-1 levels [48, 49]. The negative association reported herein between ODI and MCP-1 levels was unexpected considering that MCP-1 gene expression increases in response to hypoxia and appears to correlate with the degree of hypoxemia in adult patients with OSA [50]. PAI-1 is an inhibitor of tissue plasminogen activator and primarily functions as a suppressor of plasma fibrinolysis. PAI-1 increases in plasma are believed to play a role in the pathophysiology of endothelial dysfunction and atherothrombosis [51]. PAI-1 has been recently shown to have a strong correlation with known cardiometabolic risk factors in adults and is proposed as a biomarker for metabolic syndrome [52]. Similarly, higher PAI-1 levels have been associated with higher risk for microvascular complications in children, as well as with poorer diabetes control and hyperlipidemia in patients with type 1 diabetes [53]. In the context of OSA, higher levels of PAI-1 have been previously described in adults [54, 55]. Here, we show for the first time that obese children with OSA have higher plasma levels of PAI-1, supporting the notion that such alterations may reflect an underlying risk for vascular dysfunction, even if measures of endothelial function were not specifically acquired. Indeed, early development of endothelial dysfunction in pediatric OSA has been the subject to recent and intense research efforts which have led to the demonstration that the microvascular bed is a target of OSA [7, 8, 56–58]. Interleukin-6 is a ubiquitously expressed proinflammatory cytokine and well-established risk factor for adverse cardiovascular outcomes [59]. IL-6 signaling pathways are involved in the liver synthesis of C-reactive protein (CRP), and CRP is elevated in children with sleep-disordered breathing, whereby both IL-6 and CRP levels correlate with degree of hypoxemia and sleep disruption, independently of the degree of obesity [60]. Elevated IL-6 levels have been now repeatedly described in both adults and children with OSA [61, 62], and genetic variations in the IL-6 gene are associated with pediatric OSA and may account for the increased CRP levels seen in those children [23]. Thus, the increased IL-6 levels in the moderate-severe group of OSA children may provide a useful indicator for the presence of a more severe clinical phenotype. However, we cannot exclude the possibility that the different genomic background in this population may account for a decreased likelihood of finding elevated IL-6 plasma concentrations as recently reported in a comparison of US and Greek children [23].

Our study is the first to examine a large pediatric cohort of obese children from the community (i.e., not clinically referred children) and evaluated these children in an unbiased fashion for the presence of sleep-disordered breathing. These were therefore a priori healthy children without any preexisting conditions except for the presence of obesity. All previous studies in which the proinflammatory effects and metabolic consequences of obesity were explored consisted of symptomatic, clinically-referred obese children being evaluated for management of their obesity and with a high prevalence of OSA, precluding systematic determination of the relative contribution of OSA to the inflammatory profile of obesity [3, 18, 19, 63, 64]. As reported above, the increase in individual inflammatory markers and in the overall IS among the OSA group was independent of the degree of obesity. Furthermore, all 3 markers altered by OSA are ascribed pathophysiological roles in cardiovascular dysfunction, thereby suggesting that OSA in obese children might predispose them to a more severe cardiovascular phenotype and to earlier development of cardiovascular morbidities. Based on our previous study showing that obese children with OSA have a significantly higher proportion of abnormal endothelial function [7], more aggressive diagnostic and intervention measures appear to be warranted by the concurrent presence of obesity and symptoms of OSA. Conversely, children with milder forms of sleep-disordered breathing, that is, RDI < 3/hrTST, had lower systemic inflammatory markers, potentially justifying the expectant approach strategy as recently recommended [65].

An interesting association emerged between increased BMI and leptin levels and decreased total sleep time during the overnight PSG. Such association concurs with epidemiological studies showing that sleep loss is associated with increased obesity, increased appetite, and elevated leptin levels in adults [66], and with similar recent findings in children [67]. Of note, reduced duration is not a primary feature of OSA, as confirmed by the similar total sleep time in OSA and no-OSA children in the present study.

The strong association between prolonged hypercapnia and increased inflammation deserves comment. Obesity-hypoventilation syndrome (OHS) is a relatively infrequent condition in children that is characterized by airway obstruction and CO_2_ retention [68]. OHS is relatively underdiagnosed, and in adults it has been associated with impaired daily functioning and increased risk for diabetes and cardiovascular morbidity (including systemic and pulmonary hypertension, ischemic heart disease, and right-heart failure), as well as with higher risk of hospitalization and death [69–72]. The occurrence of alveolar hypoventilation during sleep is much more common in obese children with OSA when compared with children with OSA who are not obese [73, 74], and the present study illustrates for the first time the possibility that children with increased CO_2_ retention may represent a high risk group.

In summary, systemic inflammation is more pronounced in obese children with OSA, further buttressing the contributions of perturbed sleep and gas exchange abnormalities to the inflammatory cascade. Further studies are needed to investigate the role of PAI-1 as a marker of endothelial dysfunction and the role of hypercapnia on increased inflammation and end-organ injury in obese and nonobese children with OSA.

## Figures and Tables

**Table 1:** Antropometric measures in OSA and no-OSA obese children.

	Total (*n* = 204)	No-OSA (*n* = 129)	OSA (*n* = 75)	*P* value
Age (years)	10.8 ± 2.6	11 ± 2.4	10.4 ± 2.8	0.1
Gender (male/female)	111/93	72/57	39/36	0.6
Height (m)	1.5 ± 0.16	1.5 ± 0.16	1.46 ± 0.17	0.1
Weight (Kg)	64.3 ± 21.1	65.2 ± 20.6	62.7 ± 22.1	0.4
BMI	27.9 ± 4.3	27.9 ± 4.1	28 ± 4.6	0.8
BMI%	96.8 ± 0.6	96.7 ± 0.6	96.8 ± 0.4	0.4
Neck circumference (cm)	34.1 ± 3.8	33.9 ± 3.8	34.3 ± 3.7	0.5
Waist circumference/hip circumference	0.9 ± 0.07	0.9 ± 0.07	0.9 ± 0.07	0.6

*Data presented as mean ± SD.

**Table 2:** Polysomnographic characteristics in OSA and no-OSA obese children.

	Total (*n* = 204)	No-OSA (*n* = 129)	OSA (*n* = 75)	*P* value
AHI (/hrTST)	3.6 ± 9.5	0.6 ± 0.6	9 ± 14.2	<0.001^†^
Time in Bed (min)	479.2 ± 45.8	482.8 ± 47	473.1 ± 43.4	0.1
Total sleep time (min)	379.6 ± 70.2	384.1 ± 70.7	372 ± 69.4	0.2
Sleep Efficiency%	78.9 + 12.8	78.9 ± 12.3	78.9 ± 13.9	0.9
Number of arousals	67.3 ± 62.5	48.2 ± 32.9	99.4 ± 84.1	<0.001^†^
Arousal index (/hrTST)	11.2 ± 11.2	7.9 ± 6.1	17 ± 15.1	<0.001^†^
Respiratory disturbance index (/hrTST)	6 ± 10.6	1.4 ± 1	14 ± 14.5	<0.001^†^
Obstructive RDI (/hrTST)	5.5 ± 10.3	1 ± 0.9	13.3 ± 13.9	<0.001^†^
Central RDI (/hrTST)	0.3 ± 1	0.2 ± 0.4	0.6 ± 1.7	0.01^†^
Baseline SpO_2_ (%)	98.1 ± 1.4	98.3 ± 1.3	98 ± 1.7	0.2
Mean SpO_2_ (%)	96.4 ± 1.5	96.7 ± 1.2	96.1 ± 1.9	0.008^†^
Nadir SpO_2_ (%)	90.5 ± 5.2	91.4 ± 3.5	89.1 ± 7	0.003^†^
Time SpO_2_ < 90%	1.1 ± 7.2	0.5 ± 3.3	2.3 ± 11.4	0.1
Oxygen desaturation index (/hrTST)	2.3 ± 9	0.7 ± 1.2	5.1 ± 14.2	0.001^†^
Peak end-tidal CO_2_ (mmHg)	46.2 ± 6.9	46.1 ± 6.1	46.2 ± 8.3	0.9
Total Sleep time with end-tidal CO_2_ > 50 mmHg (hours)	3.6 ± 11.8	1.6 ± 5.6	7.1 ± 17.7	0.003^†^

^†^Statistically significant difference.

**Table 3:** Inflammatory markers in OSA and non-OSA obese children.

	Total (*n* = 204)	No-OSA (*n* = 129)	OSA (*n* = 75)	*P* value
IL-6 (pg/mL)	7.5 ± 3.8	7.3 ± 3.2	8 ± 4.8	0.2
	[7–8.1]	[6.7–7.8]	[6.8–9.1]	

IL-18 (pg/mL)	170.2 ± 96.8	163.2 ± 80.8	182.4 ± 119.2	0.17
	[156.9–183.6]	[149.1–177.2]	[155.1–209.9]	

PAI-1 (ng/mL)	3.3 ± 1.2	3.2 ± 1.2	3.6 ± 1.3	0.01^†^
	[3.1–3.5]	[2.9–3.4]	[3.3–3.9]	

MCP-1 (pg/mL)	35.1 ± 16.9	33.2 ± 15.2	38.4 ± 19.1	0.03^†^
	[32.8–37.5]	[30.6–35.9]	[34–42.8]	

Apelin C (ng/mL)	127.9 ± 118.9	125.9 ± 80.8	131.3 ± 165.8	0.7
	[111.5–144.3]	[111.9–140]	[93.1–169.4]	

Adropin (ng/mL)	0.8 ± 0.3	0.8 ± 0.3	0.87 ± 0.32	0.1
	[0.79–0.87]	[0.75–0.85]	[0.79–0.94]	

Adiponectin (*µ*g/mL)	28.1 ± 13.3	26.8 ± 12.1	30.3 ± 14.9	0.07
	[26.2–29.9]	[24.6–28.9]	[26.8–33.7]	

MMP-9 (*µ*g/mL)	0.9 ± 0.6	0.9 ± 0.5	1 ± 0.8	0.1
	[0.85–1]	[0.8–0.97]	[0.85–1.2]	

Osteocrin (ng/mL)	8.5 ± 12.6	7.8 ± 7.2	9.7 ± 18.5	0.3
	[6.7–10.2]	[6.5–9.1]	[5.5–14]	

Leptin (ng/mL)	19.1 ± 8.1	18.5 ± 8.2	20 ± 8	0.2
	[17.9–20.2]	[17.1–19.9]	[18.1–21.8]	

IS	0 ± 4.3	−0.5 ± 3.4	0.8 ± 5.4	0.04^†^
	[−0.49–4.9]	[−1.1–0.13]	[−0.43–2.1]	

*Data presented as mean ± SD [CI_95%_]. ^†^Statistically significant difference; IS: inflammatory cumulative score.

**Table 4:** Univariate associations among inflammatory markers and PSG measures in children with OSA.

Marker	Clinical variable	PCC	*P* value
MCP-1	Oxygen desaturation index	−0.276	0.017
	Nadir SpO_2_	0.232	0.02
	TCO_2_ > 50	0.412	0.001

Leptin	Total sleep time	−0.413	<0.001

Adropin	Total time in bed	−0.363	0.001
	Baseline SpO_2_	−0.471	<0.001
	TCO_2_ > 50	−0.335	0.007
	Peak CO_2_	−0.389	0.001

IL-18	Baseline SpO_2_	−0.290	0.01

MMP-9	TCO_2_ > 50	0.273	0.03
	Total time in bed	−0.310	0.007

Apelin C	TCO_2_ > 50	0.511	<0.001
